# The Hidden Burden of Fractures in People Living With HIV

**DOI:** 10.1002/jbm4.10055

**Published:** 2018-06-20

**Authors:** Melissa O Premaor, Juliet E Compston

**Affiliations:** ^1^ Department of Clinical Medicine Health Sciences Center Federal University of Santa Maria Santa Maria Brazil; ^2^ Department of Medicine Cambridge Biomedical Campus Cambridge United Kingdom

**Keywords:** Fracture, HIV, AIDS, Osteoporosis

## Abstract

The survival of people living with human immunodeficiency virus (HIV) has increased markedly since the advent of antiretroviral therapy (ART). However, other morbidities have emerged, including osteoporosis. The estimated incidence of fractures at any site in people living with HIV ranges from 0.1 per 1000 person‐years to 8.4 per 1000 person‐years: at least twice that of people without HIV. This increased risk seems to be related to HIV itself and its treatment. Risk factors for bone disease in HIV‐positive (HIV^+^) subjects include both classical risk factors for osteoporosis and fracture and factors linked to HIV itself, such as inflammation, reconstitution syndrome, low CD4, ART, and co‐infection with hepatitis B and C viruses. The risk of fractures in these individuals can be at least partially assessed by measurement of BMD and the Fracture Risk Assessment Tool (FRAX™). Only alendronate and zoledronic acid have been studied in HIV^+^ individuals; both show beneficial effects on BMD, although data on fracture reduction are not available. © 2018 The Authors. *JBMR Plus* Published by Wiley Periodicals, Inc. on behalf of American Society for Bone and Mineral Research.

## Introduction

The first cases of acquired immunodeficiency syndrome (AIDS) were described at the beginning of the 1980s. Infection with the immunodeficiency virus (HIV) presented, although varying in some cases, a typical evolution. In the early days, primary infection was characterized by a cellular and humoral immune response against the virus, followed by a prolonged period of clinical latency—in which the patient remained virtually asymptomatic—and subsequently by the appearance of clinical signs and symptoms of the disease. The mean time between the clinical onset of the disease and death was approximately 2 years.^1^ With the advent of antiretroviral therapy (ART) in the mid‐1990s, there was a dramatic decline in morbidity and mortality^2^, ^3^; currently, the life expectancy of HIV‐positive (HIV^+^) subjects who are diagnosed and treated promptly is close to that of HIV‐negative (HIV^−^) subjects.^4^, ^5^

As life expectancy in people living with HIV has increased, a number of comorbidities have become apparent; many of these are common in the general population, but develop prematurely in HIV‐infected individuals. The skeleton is one of many tissues affected by HIV infection and its treatment. Although the etiology of bone changes remains incompletely understood, prospective and cross‐sectional studies demonstrate that HIV^+^ patients have lower BMD and increased fracture risk compared with the general population.^6^, ^7^, ^8^, ^9^, ^10^, ^11^, ^12^, ^13^, ^14^, ^15^, ^16^, ^17^, ^18^, ^19^, ^20^, ^21^, ^22^, ^23^, ^24^, ^25^, ^26^, ^27^, ^28^, ^29^, ^30^, ^31^, ^32^ The aim of this review is to describe what is known about the epidemiology, pathogenesis, pathophysiology, and management of bone disease in people living with HIV.

## The Hidden Burden of Fractures in HIV‐Positive Subjects

An increase in the incidence of fractures in HIV^+^ individuals was initially reported at the end of the last decade. Since then, several studies have described the incidence of fractures in people living with HIV (Table 1).^6^, ^7^, ^8^, ^9^, ^10^, ^11^, ^12^, ^13^, ^14^, ^15^, ^16^, ^17^, ^18^, ^19^, ^20^, ^21^, ^22^, ^23^, ^24^, ^25^, ^26^, ^27^, ^28^, ^29^, ^30^, ^31^, ^32^

**Table 1:** Characteristics of the Studies That Evaluated Fracture Frequency and Fracture Risk in People Living With HIV

Author	Year	Study design	Site	Age (years)	Male gender (%)	HAART (%)	Outcome
Arnsten et al.^6^	2007	Cohort	USA	55	100	–	Fracture incidence
Battalora et al.^7^	2016	Cohort	USA	43 (36–49)	83.2	96.1	Fracture incidence
Bedimo et al.^8^	2012	Cohort	USA	18–70+	98	69.4	Fracture incidence
Borges et al.^9^	2017	Cohort	Europe, Argentina, Israel	41	75	90	Fracture incidence
Collin et al.^10^	2009	Cohort	France	36	77.2	100	Fracture incidence
Gallant et al.^11^	2004	RCT	South America, Europe, USA	36	73.9	100	Fracture incidence
Gedmintas et al.^12^	2017	Cohort	USA	43	72	100	Fracture incidence
Guaraldi et al.^13^	2011	Case‐control	Italy	46	63	–	Risk of fracture
Güerri‐Fernandez et al.^14^	2013	Cohort	Spain	50	75.3	–	Risk of fracture
Hansen et al.^15^	2012	Cohort	Denmark	37(31–45)	76	78	Risk of fracture
Hasse et al.^16^	2011	Cohort	Swiss	45 (39–51)	70.8	85.1	Fracture incidence
Kurita et al.^17^	2014	Cohort	Japan	15–81	92.8	65.9	Fracture incidence
Mazzotta et al.^19^	2015	Cross‐sectional	Italy	44	70.6	79.7	Fracture prevalence
Martin et al.^18^	2009	RCT	Australia	45	97.5	100	Major fracture incidence
Mundy et al.^20^	2012	Case‐control	USA	40	71	50	Risk of fractures in HIV‐treated people
Peters et al.^21^	2013	Case‐control	UK	46	60	85	Risk of fractures
Prieto‐Alhambra et al.^22^	2014	Case‐control	Denmark	43	48.2	–	Risk of fractures
Prior et al.^23^	2007	Case‐control	Canada	38	100	72.5	Risk of fractures
Sharma et al.^24^	2015	Cohort	USA	40 (34–46)	0	63	Risk of fractures
Short et al.^25^	2014	Cross‐sectional	UK	45 (38–51)	100	78	Fracture prevalence
Triant et al.^26^	2008	Case‐control	USA	20–79	65.2	–	Risk of fractures
Womack et al.^27^	2011	Cohort	USA	53 (48–61)	100	75	Increased risk of fractures
Yang et al.^28^	2012	Cohort	Taiwan	<20–>60	76.9–90.1	–	Orthopedic injury incidence
Yin et al.^29^	2012	Cohort	USA	39 (33–45)	83	99.7	Fracture incidence
Yin et al.^30^	2010	Cross‐sectional	USA	56	0	79.3	Prevalence of fractures
Yong et al.^31^	2011	Case‐control	Australia	49.8	88.5	80.3	Fracture incidence
Young et al.^32^	2011	Cohort	USA	40 (34–46)	79	72.7	Fracture incidence

HIV = human immunodeficiency virus; HAART = highly active antiretroviral therapy; – = information not given; RCT = randomized clinical trial; ICD = International Code of Diseases.

**Figure 1:**
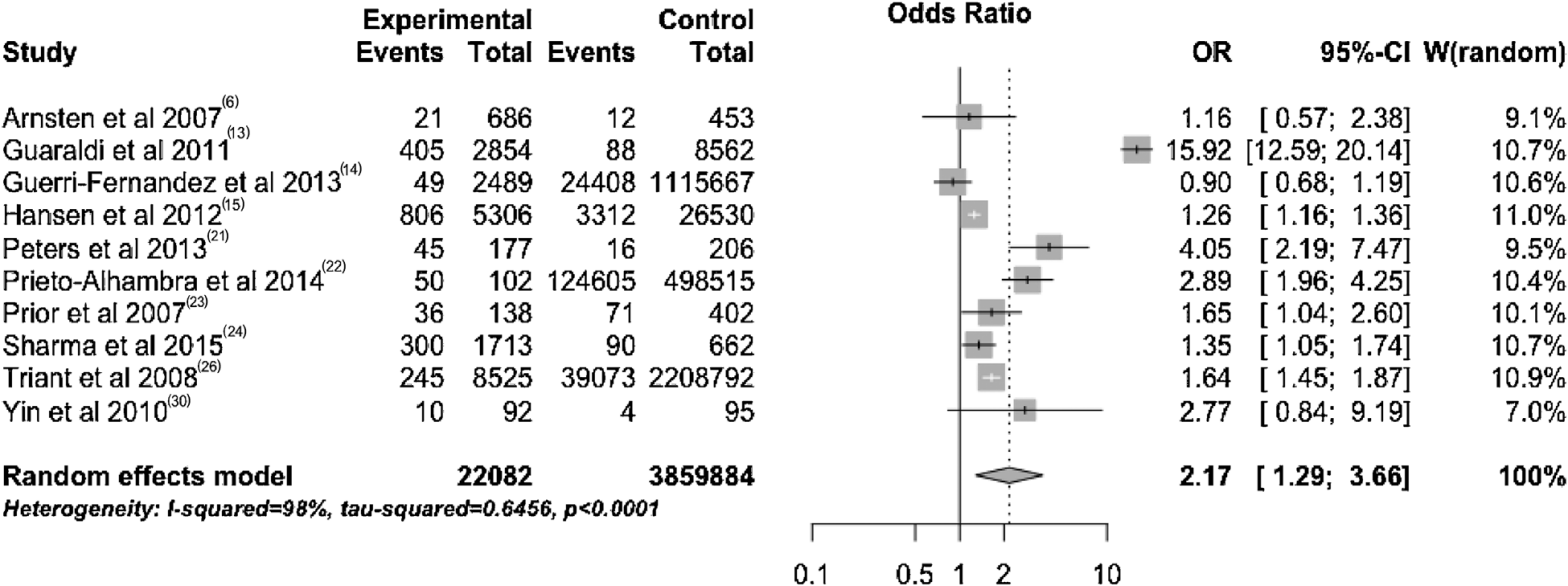
Forest plot of the odds ratio of total fractures in HIV‐positive subjects.

The reported incidence of fractures at any skeletal site in people living with HIV ranges from 0.1 fractures per 1000 person‐years to 8.4 fractures per 1000 person‐years (Table 2).^6^, ^7^, ^8^, ^9^, ^10^, ^11^, ^12^, ^14^, ^15^, ^16^, ^17^, ^29^, ^31^, ^32^ However, it is important to note that most of the studies were carried out in men with a mean age of between 36 and 56 years (Table 1).^6^, ^7^, ^8^, ^9^, ^10^, ^11^, ^12^, ^13^, ^14^, ^15^, ^16^, ^17^, ^18^, ^19^, ^20^, ^21^, ^22^, ^23^, ^24^, ^25^, ^26^, ^27^, ^28^, ^29^, ^30^, ^31^, ^32^ Men in this age group usually have a low frequency of fractures, so comparison with an age and sex‐matched population is important. Shiau and colleagues conducted a systematic review and meta‐analysis in 2012.^33^ They found a crude incidence ratio of 1.58 (95% CI, 1.25 to 2.00) for fracture at any site in HIV^+^ individuals when compared with HIV^−^ controls.^33^ As the aim of Shiau et al.'s meta‐analysis was to evaluate the incidence of fractures and not the risk of fractures, they included only cohort studies. Furthermore, after this meta‐analysis,^33^ more studies have been published.^14^, ^21^, ^22^ Our group has also performed a new meta‐analysis to evaluate the risk of fractures in people living with HIV for this review (see Supporting Information online for protocol), which included 10 studies. The odds ratio (OR) for fracture in people living with HIV was 2.17 (95% CI, 1.29 to 3.66; Fig. 1).

**Table 2:** Incidence of Fracture at Any Site by Cohort

Cohort	Incident fracture^a^	95% CI
Arnsten et al., 2007^6^	3.1	1.9–4.6
Battalora et al., 2016^7^	8.4	6.8–10.3
Bedimo et al., 2012^8^	0.3	0.3–0.3
Borges et al., 2017^9^	4.2	3.8–4.6
Collin et al., 2009^10^	0.3	0.1–0.9
Gallant et al., 2004^11^	1	0.6–1.7
Gedmintas et al., 2017^12^	2.2	1.9–2.5
Guerri‐Fernandez et al., 2013^14^	0.8	0.3–1.6
Hansen et al., 2012^15^	2.1	2.0–2.2
Hasse et al., 2011^16^	0.7	0.6–0.8
Kurita et al., 2014^17^	0.1	0.0–0.3
Yin et al., 2012^29^	0.1	0.1–0.1
Yong et al., 2011^31^	0.5	0.4–1.6
Young et al., 2011^32^	0.3	0.2–0.3

^a^ Per 1000 persons/years.

Recent studies have also reported an increased risk of vertebral fractures in HIV+ subjects.^24^, ^27^, ^30^, ^31^, ^32^, ^34^ In a systematic review and meta‐analysis, Ilha et al. reported that the prevalence of vertebral fractures was 11.1% (95% CI, 4.5 to 25.0).^35^ As in HIV^−^ individuals, the prevalence of clinical vertebral fractures was lower, 3.9% (95% CI, 0.9 to 15.8) than the prevalence of morphometric vertebral fractures, 20.2% (95% CI, 15.7 to 25.6), when evaluated by X‐ray.^35^ These findings suggest that, as in the general population, most spine fractures in people living with HIV do not come to clinical attention. The risk of vertebral fractures in these individuals was 2.30 times greater than the corresponding risk in the general population (OR = 2.30; 95% CI, 1.37 to 3.85).^35^

## Bone Mineral Density and Bone Quality in HIV‐Positive Subjects

Low BMD in people living with HIV has been consistently reported.^36^, ^37^, ^38^ The prevalence of osteoporosis in both the lumbar spine and the hip is twice as high as in people living without HIV.^38^ Furthermore, in HIV^+^ individuals treated with ART, this frequency increases by threefold when compared with HIV^−^ controls.^38^

Although low BMD is well documented, little is known about the effects of HIV on bone microstructure and bone quality. Changes in trabecular and cortical bone structure have been described in premenopausal^39^ and postmenopausal women,^40^ as well as in young^41^ and middle‐aged men.^42^ Calmi and colleagues found a decrease in the trabecular number and trabecular density in the tibial bone of HIV^+^ women using ART when compared with HIV^−^ women.^39^ They also found a decrease in the cortical density of the radius of HIV^+^ women when compared with controls.^39^ In postmenopausal African American and Hispanic women, the cortical area and thickness of the tibia were approximately 12% lower in HIV^+^ than in HIV^−^ individuals.^40^ Another study in HIV^+^ young men infected in childhood or adolescence found similar results, with a decrease in cortical and trabecular thickness of the radius and tibia when compared with HIV^−^ men of the same age.^41^ Similarly, Tan and colleagues described a decrease in cortical thickness of the tibia and radius in HIV^+^ middle‐aged men.^42^

Bone quality is difficult to assess in clinical studies; therefore, such data are scarce. Guerri‐Fernandez and colleagues used the microindentation technique to evaluate the material properties of bone at the tissue level in 50 HIV^+^ individuals with a mean age of approximately 37 years.^43^ These individuals had a lower bone material strength index (BMSi) when compared with matched controls.^43^ Yin and colleagues evaluated bone strength in young men using finite element analysis^41^ and reported a 14% to 17% decrease in bone stiffness in HIV^+^ subjects.^41^

## Bone and Antiretroviral Therapy

The initiation of ART is associated with a loss of 2% to 6% of BMD at the hip and the spine.^44^, ^45^, ^46^, ^47^, ^48^, ^49^ This bone loss appears to stabilize at 24 months after antiretroviral initiation,^44^, ^45^, ^48^ with a subsequent plateau or even increase after this period.^44^, ^45^, ^48^ The longer‐term effects of ART are unknown because the longest follow‐up extends to only 72 months.^38^ Different forms of ART vary in their effects on bone. Regimens, including a nucleotide reverse transcriptase inhibitor (TDF) and protease inhibitors, have been described as the most deleterious.^8^, ^9^ On the other hand, the new integrase inhibitors seem to have a better bone profile.^45^, ^50^, ^51^, ^52^ Most studies describe a low rate of bone loss, or even bone gain, with raltegravir or dolutegravir.^45^, ^50^, ^51^, ^52^

Even within the same class of antiretroviral drugs the effect on bone may vary. A newer form of tenofovir, tenofovir alafenamide, has been associated with lower BMD loss than with TDF.^53^ Furthermore, in the ASSERT study (Study of Once‐Daily Abacavir/Lamivudine Versus Tenofovir/Emtricitabine, Administered With Efavirenz in Antiretroviral‐Naive, HIV‐1 Infected Adult Subjects) a greater decrease in BMD was shown in subjects treated with tenofovir–emtricitabine when compared with abacavir–lamivudine after 96 weeks of treatment.^44^ It is important to note, however, that both ART regimens were associated with bone loss.^44^ Recently, Guerri‐Fernandez and colleagues reported lower BMD in long‐term users of tenofovir compared with long‐term users of abacavir in a small cross‐sectional study.^54^ Nonetheless, they found no differences in trabecular bone score (TBS) or microindentation‐derived BMSi between the two groups.^54^

At present, the only associations reported between specific forms of ART and fracture risk are for TDF and protease inhibitors.^8^, ^9^ It is important to note that most of the studies that evaluated the incidence of fractures in people living with HIV had insufficient power to evaluate the association between specific antiretrovirals and fractures.

## Bone Turnover Markers in HIV‐Positive Subjects

Changes in bone markers have been described in several studies in HIV^+^ individuals.^44^, ^45^, ^52^, ^55^ The initiation of ART is associated with an increase in markers of both bone resorption and bone formation^44^, ^45^, ^52^; in prospective studies, this increase has been associated with a decrease in BMD.^44^, ^45^

The magnitude of the rise in markers of bone formation and resorption varies according to the ART used^45^, ^52^ and is greater in combinations that include TDF.^45^ The increase in turnover markers peaks at 48 weeks after initiation of ART,^44^, ^52^ decreasing or stabilizing afterwards.^52^ This time course is similar to that seen for immune reconstitution, as described by Ofotokun and colleagues.^55^

The SMART Body Composition substudy, an arm of the Strategies for Management of Antiretroviral Therapy (SMART) trial, reported lower serum levels of bone‐specific alkaline phosphatase (bALP), osteocalcin, N‐terminal propeptide of type 1 procollagen (P1NP), and C‐terminal telopeptide of type 1 collagen (CTx) in individuals that received intermittent ART compared with those who received continuous ART at the end of 12 months.^56^

Although current evidence suggests that the effect of ART on bone turnover markers may become attenuated with longer duration of therapy,^52^ little is known about longer‐term changes. In the study by Yin and colleagues, which included African American and Hispanic postmenopausal women, elevated levels of bone markers were present even in those who had taken ART for more than 4 years.^30^

Because of the proven efficacy of ART in increasing the survival of HIV^+^ individuals, there have been few studies in treatment‐naive individuals. Moreover, the studies that have been performed in untreated subjects have mainly included those with a low or undetectable viral load, making it difficult to assess the impact of HIV per se on bone metabolism. In a study carried out in 1995, a histomorphometric analysis of iliac crest biopsy samples from 22 untreated HIV^+^ adults showed a significant reduction in bone turnover.^57^ In another small study of treatment‐naïve young subjects, values for bone resorption and formation markers were within those expected for the general population.^58^ Likewise, in postmenopausal HIV^+^ women without treatment in the Yin et al. study, bone markers were at levels similar to those in HIV^−^ controls.^30^

## Factors Associated With Bone Metabolic Disease in HIV Subjects

Many risk factors for osteoporosis and fracture have been identified in HIV^+^ subjects. Some of these are common to the general population, whereas others are related to HIV itself (Table 3).

**Table 3:** Factors Associated With Bone Disease and Fracture in People Living With HIV

Factors in common with the general population	Factors related to the HIV
Aging^6^, ^32^, ^38^	Chronic inflammation^55^
Previous fractures^38^, ^59^	Reconstitution syndrome^55^
Low BMI^32^, ^37^, ^60^, ^61^	ART use^8^, ^15^, ^20^, ^27^
Tobacco use^38^, ^62^	Co‐infection with hepatitis B^79^
Alcohol abuse^63^	Co‐infection with hepatitis C^33^, ^80^, ^81^
Glucocorticoid use^63^	Low CD4^31^, ^32^, ^38^, ^81^
Anticonvulsant use^59^	AIDS‐defining disease^81^
Postmenopausal status^6^, ^24^, ^26^	
Hypogonadism^38^, ^64^	
Vitamin D deficiency^24^, ^65^, ^66^, ^67^	
White race^8^, ^27^	
Diabetes mellitus^59^	
Frailty^69^	
Sarcopenia^38^	
Selective serotonin reuptake inhibitors^70^	
Comorbidities^72^	
Falls^59^	
Renal disease^59^	

HIV = human immunodeficiency virus; AIDS = acquired immunodeficiency syndrome; ART = antiretroviral therapy.

The presence of previous fractures,^38^, ^59^ a low BMI,^32^, ^37^, ^60^, ^61^ tobacco^38^, ^62^ and alcohol abuse,^63^ the use of glucocorticoids,^63^ the use of anticonvulsants,^59^ postmenopausal status,^6^, ^24^, ^26^ hypogonadism,^38^, ^64^ vitamin D deficiency,^24^, ^65^, ^66^, ^67^ white race,^8^, ^27^ renal disease,^59^, ^68^ falls,^59^ diabetes mellitus (DM),^59^ and aging^6^, ^32^, ^38^ are factors classically associated with fragility fracture and have also been described in HIV^+^ subjects. Other factors most recently associated with an increased risk of fractures in non‐HIV subjects such as frailty,^69^ sarcopenia,^38^ selective serotonin reuptake inhibitors (SSRIs),^70^ cardiovascular disease,^68^ cancer,^68^ liver disease,^68^ neurocognitive impairment,^71^ and other comorbidities^72^ have also been reported in HIV^+^ individuals.

Some of the risk factors commonly present in the general population appear to have increased frequency in people living with HIV. The incidence of type 2 DM is at least 1.4 times higher in this population, and it seems to occur at an earlier age when compared with the general population.^73^, ^74^ In addition, HIV^+^ men may have low testosterone levels,^75^ and the frequency of hypogonadism is increased in people living with HIV.^64^, ^76^ There is also an increased prevalence of vitamin D deficiency when compared to the general population,^65^, ^66^ possibly due to the presence of several risk factors for vitamin D deficiency, including the use of efavirenz (a non‐nucleoside reverse transcriptase inhibitor)^77^ or protease inhibitors,^65^, ^78^ anticonvulsant therapy, kidney disease, and liver disease.^66^

Factors specifically associated with HIV are the presence of chronic inflammation,^55^ reconstitution syndrome,^55^ the use of ART,^8^, ^15^, ^20^, ^27^ co‐infection with hepatitis B^79^ or C,^33^, ^80^, ^81^ low CD4,^31^, ^32^, ^38^, ^81^ or an AIDS‐defining disease.^81^ Co‐infection with hepatitis C virus is associated with a one‐ to twofold increase in the risk of fractures when compared with monoinfection with HIV.^80^ Similarly, treatment for co‐infection with hepatitis B virus appears to increase the risk of fractures in these individuals.^79^ CD4 counts below 200 cells/mm^3^ have been associated with a higher incidence of fractures in HIV^+^ subjects in cohort studies.^31^, ^32^

Other factors, such as cocaine use, injectable drug abuse, the use of opioids, methadone maintenance therapy, hyperparathyroidism, and growth hormone deficiency, have also been associated with fractures in HIV^+^ individuals.^59^, ^63^, ^72^

People living with HIV have an increased risk both of fragility fractures and traumatic fractures. The latter may be associated with factors such as substance abuse, high‐risk behavior increasing the risk of physical injury, and neurocognitive dysfunction, among others.^68^, ^71^

## Mechanisms of Bone Disease in HIV‐Positive Subjects

The factors responsible for bone disease in HIV^+^ individuals have not been fully established. Chronic inflammation activates inflammatory cytokines and TNF alpha, resulting in stimulation of RANKL production and increased bone resorption. Hilleman and colleagues suggest that the dysregulation of B cells present in these individuals may also contribute to bone loss,^72^, ^82^ postulating an imbalance between B‐lymphocytic expression of RANKL and osteoprotegerin. Furthermore, the imbalance that occurs in the immune reconstitution syndrome may also contribute to bone loss in HIV subjects soon after ART initiation.^72^, ^82^

Little is known about drug‐specific mechanisms of antiretroviral bone loss. TDF is associated with the increased renal loss of phosphate and secondary hyperparathyroidism.^70^, ^83^, ^84^ In extreme cases, Fanconi's syndrome has been described.^85^ As mentioned earlier, efavirenz and protease inhibitors affect vitamin D metabolism.(78,86, 87, 88) Efavirenz acts on cytochrome P450 enzymes, decreasing the expression of CYP2R1 enzyme and increasing the expression of CYP24, which plays a role in the conversion of vitamin D to 25‐ hydroxyvitamin D [25(OH)D] and to inactive metabolites.^86^, ^87^, ^88^ In addition, protease inhibitors inhibit the action of 25‐hydroxylase and 1‐alpha‐hydroxylase enzymes.^78^, ^86^

It is speculated that HIV itself might be associated with some bone toxicity. Although there is no evidence of infection of bone cells by the virus, some viral proteins appear to have a negative interaction with bone metabolism.^89^, ^90^, ^91^ The HIV‐1 gp 120, and the Tat, Nef, Rev, and Vpr proteins have been shown to have some adverse effects on bone in experimental studies.^89^, ^90^, ^91^, ^92^, ^93^ The HIV gp 120 protein appears to interact negatively with osteoblasts, stimulating their apoptosis.^89^, ^90^, ^91^, ^92^, ^94^ This protein also appears to reduce the activity of alkaline phosphatase, reduce bone mineralization, and interfere with the expression of runt‐related transcription factor 2 (RUNX‐2).^91^ Tat and Nef proteins decrease the number of progenitor cells in the bone marrow with the potential to differentiate into osteoblasts.^93^ Vpr protein increases RANKL expression in peripheral blood monocytes,^91^ whereas Tat and Rev proteins divert the differentiation of these monocytes towards osteoclasts.^94^

## Fracture Risk Assessment in HIV‐Positive Subjects

As in the general population, the risk of fractures in people living with HIV increases as BMD decreases. There is a threefold increase in the risk of fractures in middle‐aged HIV^+^ men with a BMD *T‐*score ≤1.^6^ In an analysis of the HIV Outpatient Study (HOPS) and Study to Understand the Natural History of HIV/AIDS (SUN Study), Batalora and colleagues found that HIV^+^ subjects with osteoporosis had a HR of 2.4 (95% CI, 2:02 to 8:01) of suffering a fracture compared with HIV^−^ subjects with normal BMD.^7^

The estimation of fracture probability in people living with HIV using the FRAX (Fracture Risk Assessment Tool) algorithm has been evaluated in several studies. This tool appears to underestimate the risk of fracture in HIV^+^ subjects. In the Veterans Aging Study Virtual Cohort (VACS‐VC), which included 24,451 men over 50 years of age, FRAX was less accurate in predicting fractures in HIV^+^ individuals when compared with HIV^−^ individuals.^95^ In this study, the FRAX algorithm was modified and the variables secondary osteoporosis and family history of hip fracture were not included, which may have contributed to the underperformance of FRAX. However, when the HIV^+^ variable was included in the calculation as a cause of secondary osteoporosis, the accuracy of the instrument was increased.^95^

The utility of TBS in fracture prediction in people living with HIV has been assessed in one study. Ciullini and colleagues^34^ studied the association between vertebral fractures and TBS in 141 HIV^+^ individuals. The subjects in the lowest quartile of TBS had a higher prevalence of vertebral fractures.^34^ This observation is interesting because BMD appears to underestimate the incidence of vertebral fractures in the same way as in non‐HIV subjects.^61^

Based on the currently available evidence, both the European AIDS Clinical Society (EACS)^96^ and the Osteo Renal Exchange Program (OREP)^97^ recommend that fracture risk assessment should be performed in all HIV‐infected individuals over 40 years of age using the FRAX algorithm. According to the EACS, those individuals with an estimated 10‐year probability of major osteoporotic fractures ≥20% should undergo BMD measurement. On the other hand, OREP recommends measuring BMD in all HIV^+^ individuals with an estimated risk of major osteoporotic fractures ≥10%.^96^, ^97^ Furthermore, the EACS recommends that in individuals with other risk factors added to HIV—postmenopausal women, men older than 50 years, previous fracture, major risk of falls, clinical hypogonadism, and the use of more than 5 mg/day of oral glucocorticoids for more than 3 months—a DXA scan should be performed.^96^

Vertebral fracture assessment is recommended by the EACS in those subjects with low bone mass at the spine or osteoporosis at any site, loss of height, or kyphosis. It can be performed with a lateral X‐ray or a bone densitometry scan.^96^ Due to the recent data showing the increased risk of spine fractures in people living with HIV, vertebral fracture assessment should be considered in high‐risk subjects.

## Morbidity and Mortality of Bone Disease in HIV Subjects

Little is known about the real impact of bone disease in people living with HIV. Fractures are associated with a poorer quality of life, loss of independence, hospitalization, and increased mortality in the general population. In a study conducted in Taiwan using data from the National Health Insurance (NHI) Program, which covers about 98% of the population, approximately 80% of fractures in HIV^+^ people required surgical intervention.^28^ There are no studies on mortality and quality of life in HIV^+^ individuals after a fracture. Nonetheless, just as in HIV^−^ subjects, HIV^+^ individuals who suffer a fracture are at increased risk of a further fracture.^38^

Although there are no studies on the impact of vertebral fractures in people living with HIV, these are associated with worsening of quality of life, functional limitation, and reduction of lung capacity in the general population. Previous vertebral fractures are also a strong risk factor for future fractures.

## Management

As in the general population, the management of osteoporosis and fracture prevention in individuals living with HIV should include a consideration of nonpharmacological and pharmacological measures. A healthy lifestyle should be recommended and a falls risk assessment should be performed and preventive measures taken when appropriate.

Although not studied in HIV^+^ individuals, an assessment of the risk of falls and measures to prevent them may be beneficial. Avoiding alcohol abuse decreases the risk of falls and also has beneficial effects on bone.^98^ Smoking cessation should be recommended in all individuals. A diet rich in calcium, fruits, and vegetables has been associated with a lower risk of fractures in the general population,^99^, ^100^ and should be recommended for people living with HIV. In addition to a higher fracture risk, these individuals are at increased risk of developing diabetes.^73^, ^74^ Therefore, the recommendation of a balanced diet can have multiple benefits in these subjects.

Studies addressing the effect of weight‐bearing exercise on HIV^+^ subjects are scarce. Santos and colleagues studied the effect of strength training in 20 HIV^+^ ART‐treated individuals with lipodystrophy.^101^ They found an increase in BMD after 12 weeks of training.^101^ These findings, in conjunction with the evidence found in the general population,^98^ corroborate the recommendation of physical exercise in these subjects.

People living with HIV are at increased risk of vitamin D deficiency. The measurement of serum 25‐hydroxyvitamin D [25(OH)D] levels should be performed in high‐risk HIV^+^ individuals. In subjects with serum levels below 20 ng/mL, the EACS recommends measurement of serum parathyroid hormone (PTH), calcium, phosphorus, and alkaline phosphatase levels.^96^ In vitamin D‐deficient individuals, vitamin D replacement with a loading dose of 10,000 IU/day for 8 to 10 weeks is recommended, followed by a maintenance dose of 800 to 2000 IU/day after achieving the goal of treatment.^96^ The recommended target serum level of 25(OH)D is over 20 ng/mL, with normalization of serum PTH levels. In addition, they recommend calcium supplementation in subjects with inadequate dietary intake.^96^

Other causes of secondary osteoporosis should be excluded.^97^ As the presence of hypogonadism is common in men living with HIV, it should be assessed with a thorough clinical history and careful physical examination. In those whom clinical evaluation suggests the presence of hypogonadism, further investigations should be performed and, if indicated, treatment established.

An early diagnosis of HIV with immediate initiation of ART may help to reduce bone disease, although this remains to be tested. In people at high risk of fracture, it may be prudent to avoid ART regimens containing TDF. Although switching to antiretroviral drugs, such as integrase inhibitors and tenofovir alafenamide, reduces adverse skeletal effects, the long‐term effects of this approach on viral resistance is not known.^44^, ^45^, ^50^, ^51^, ^52^, ^53^, ^54^ For this reason, any change in ART should be evaluated in conjunction with the patient's HIV treatment team.

Both alendronate and zoledronic acid are effective in reducing bone loss in people living with HIV.^102^, ^103^, ^104^, ^105^, ^106^, ^107^, ^108^, ^109^, ^110^ In a meta‐analysis conducted by Pinzone and colleagues, there was less of a decrease in bone mass in both the hip and lumbar spine of HIV^+^ individuals treated with bisphosphonates when compared with HIV^+^ individuals receiving placebo.^111^ The mean difference between the groups after 96 months of treatment was 6.76% (95% CI, 4.98 to 8.54) in the lumbar spine and 3.2% (95% CI, 1.52 to 4.88) in the hip.^111^ Although there are no studies on the antifracture efficacy of these drugs, extrapolation from evidence for their effects in the general population appears reasonable. Some studies using zoledronic acid suggest that BMD gains are maintained with administration at approximately two yearly intervals.^107^, ^110^ In another study, Bolland and colleagues studied the effect of zoledronic acid on 35 HIV^+^ subjects using ART 5 years after the second dose of the drug. They found a persistence of beneficial effects on bone mass without evidence of significant adverse effects in these individuals.^107^

There are no studies evaluating the effects of denosumab in HIV^+^ people. Some authors report theoretical concerns about an increased risk of infections associated with this drug.^112^ However, concerns about the risk of serious infections associated with denosumab therapy have not been confirmed in the general population.^113^ Furthermore, denosumab has been used in other populations that are considered to be immunosuppressed, for example, subjects with rheumatoid arthritis or cancer, without an increase in risk of infections.^114^, ^115^ The use of bone‐forming medications in people living with HIV has also not been studied, although there is one case report describing the use of teriparatide in a 70‐year‐old HIV^+^ individual with a vertebral fracture.^116^ Thus, at present, bisphosphonates provide the first‐line treatment of HIV^+^ subjects at increased risk of fracture; in those with contraindications or intolerance, teriparatide and denosumab provide alternatives. Adherence to alendronate is poor, with up to 60% of women stopping this drug after one year^117^, ^118^; as zoledronic acid is administered once yearly by intravenous infusion, it may be the preferred option.

## Conclusion

People living with HIV have a fracture risk twice that of people without HIV. The pathogenesis of increased bone fragility is multifactorial and includes both traditional and HIV‐specific risk factors. Fracture risk should be assessed in HIV^+^ individuals using clinical risk factors and, where indicated, measurement of BMD. Lifestyle measures to optimize bone health should be recommended in all people living with HIV; bisphosphonates are the first‐line treatment of those at increased fracture risk. Further studies are required to establish whether early identification of HIV^+^ individuals and prompt initiation of ART reduces the risk of bone disease.

### Disclosures

All authors state that they have no conflict of interest regarding this manuscript. JEC has received advisory and speaking fees from Gilead and speaking fees from Amgen.

## Supporting information

Supporting Appendix S1.
