# Supplementary material for: The Hidden Burden of Fractures in People Living With HIV
Source: JBMR Plus. 2018 Jun 20;2(5):247–56. doi: 10.1002/jbm4.10055 (PMC6139727; doi:10.1002/jbm4.10055)
Supplement: Supplementary file 1 — Supporting Appendix S1. [file JBM4-2-247-s001.docx]

**Appendix**

**META-ANALYSIS PROTOCOL**

The meta-analysis was carried out according to the PRISMA
Guidelines^(1)^. The Research Committee of Health Sciences Centre of the Federal University of Santa Maria approved the study protocol (045911). The PRISMA flowchart is displayed at Figure 2.

Studies were included in the meta-analysis if they met the following criteria: (1) Randomized controlled trials, cohort studies, cross-sectional, and case-control; (2) men and women aged over 18 years, HIV-positive with or without antiretroviral therapy; (3) have evaluated the risk of a fracture that occurred at any site. Animal studies, studies that evaluated specific cohorts of patients with HIV (for example, studies that assess a particular variable only in patients e.g. HIV with hepatitis-C, or just HIV with lipodystrophy), or those that did not meet the inclusion criteria were excluded from the meta-analysis.

The search for studies was performed in EMBASE (Elsevier), PubMed, the Regional Library of Medicine (BIREME), and the Cochrane Library (Cochrane Database of Systematic Reviews - CDSR). Also, studies based on the reference lists of the included articles were analysed. Studies written in any language and with no publication date limits were considered.

The terms used include the Descriptors in Health Sciences (DeCS), MeSH and Emtree (EMBASE) terms, which were modified for each database. The terms used were: ("hiv" [MeSH Terms] OR "hiv" [All Fields]) AND ("spinal fractures" [MeSH Terms] OR "fracture"[All Fields]) in Pubmed; (tw:(Fractures, Bone)) AND (tw:( HIV)OR (tw:(Acquired Immunodeficiency Syndrome)OR (tw:(Anti-Retroviral Agents)), in Bireme; 1 'human immunodeficiency virus' OR 'antiretrovirus agent' OR 'acquired immunodeficiency' OR 'acquired immunodeficiency syndrome' AND 2 'fracture'/exp OR 'fracture' OR 'fragility fracture'/exp OR 'fragility fracture' OR AND 3 'human'/de AND ([adult]/lim OR [aged]/lim OR [middle aged]/lim OR [very elderly]/lim OR [young adult]/lim) in EMBASE; and "fracture":ti,ab,kw) AND ("HIV positive":ti,ab,kw OR "AIDS":ti,ab,kw OR "antiretroviral therapies":ti,ab,kw), restricted only to clinical trial and cohort studies in COCHRANE.

Whenever different articles from the same database were obtained, only the most complete was included in the meta-analysis.

**Selection process**

Two protocol members performed the selection of the studies independently. Firstly, the studies were screened based on their titles and abstracts. The studies that could not be ruled out in this procedure had their full texts evaluated. Additionally, for all selected items, the full texts were sought, and their eligibility was double-checked. If there was disagreement between the two reviewers regarding the identification, eligibility, and inclusion of items, they discuss the article to reach a consensus.

**Data collection process**

Two protocol members extracted the data of each study, independently. The agreement between the two extractors should be 100%. In the cases where there was disagreement they discuss the article to reach a consensus. The following data were extracted from each article: the name of the first author, year of publication, study design, site, age, gender, ART use, number of total fractures.

**Risk of bias (quality) assessment**

The bias risk assessment was conducted by the Newcastle-Ottawa scale ^(2)^. All included studies were cohort, case–control, or cross-sectional studies. The cross-sectional study was evaluated as a case-control study. The Newcastle-Ottawa scale assessed the selection, comparability and exposure of a case-control study and selection, comparability, and outcome of a cohort study. In it, 9 stars represent maximum score for a study, and the study with over 6 stars would be regarded as relatively high quality. The quality of the included studies is presented in Table 4.

**Data synthesis and statistical analysis**

The risk of total fractures was summarized as odds ratio. We pooled the odds ratio using the Mantel-Haenszel method. We have used the random effects model, with DerSimonian and Laird as variance estimator. The statistical heterogeneity among studies was assessed using Cochran’s Q test and the inconsistency I2 test. We performed an additional analysis with fixed-effects model in order to qualitatively evaluate differences in point estimates provided by models with random and fixed effects. Both models were similar [Random effect model: OR 1.72 (95% CI 1.62 to 1.820]. Publication bias was evaluated using a funnel plot; the Begg test was used as a statistical parameter for testing funnel asymmetry (Figure 3). All the analyses were made using the software R [R version 3.2.4, 2016, The R Foundation for Statistical Computing, Platform: x86_64-apple-darwin13.4.0 (64-bit)] and RStudio [RStudio Team (2015). RStudio: Integrated Development for R. RStudio, Inc., Boston, MA URL <http://www.rstudio.com/>.

**References**

1. Hutton B, Salanti G, Caldwell DM, Chaimani A, Schmid CH, Cameron C, et al. The PRISMA extension statement for reporting of systematic reviews incorporating network meta-analyses of health care interventions: checklist and explanations. Ann Intern Med. Jun 02 2015;162(11):777-84. Epub 2015/06/02.

2. Stang A. Critical evaluation of the Newcastle-Ottawa scale for the assessment of the quality of nonrandomized studies in meta-analyses. Eur J Epidemiol. Sep 2010;25(9):603-5. Epub 2010/07/24.

3. Arnsten JH, Freeman R, Howard AA, Floris-Moore M, Lo Y, Klein RS. Decreased bone mineral density and increased fracture risk in aging men with or at risk for HIV infection. AIDS. 2007;21(5):617-23.

4. Guaraldi G, Orlando G, Zona S, Menozzi M, Carli F, Garlassi E, et al. Premature age-related comorbidities among HIV-infected persons compared with the general population. Clinical Infectious Diseases. 2011;53(11):1120-6.

5. Güerri-Fernandez R, Vestergaard P, Carbonell C, Knobel H, Avilés FF, Castro AS, et al. HIV infection is strongly associated with hip fracture risk, independently of age, gender, and comorbidities: A population-based cohort study. Journal of Bone and Mineral Research. 2013;28(6):1259-63.

6. Hansen ABE, Gerstoft J, Kronborg G, Larsen CS, Pedersen C, Pedersen G, et al. Incidence of low and high-energy fractures in persons with and without HIV infection: A Danish population-based cohort study. AIDS. 2012;26(3):285-93.

7. Peters BS, Perry M, Wierzbicki AS, Wolber LE, Blake GM, Patel N, et al. A cross-sectional randomised study of fracture risk in people with HIV infection in the probono 1 study. PLoS One. 2013;8(10).

8. Prieto-Alhambra D, Güerri-Fernández R, De Vries F, Lalmohamed A, Bazelier M, Starup-Linde J, et al. HIV infection and its association with an excess risk of clinical fractures: A nationwide case-control study. Journal of Acquired Immune Deficiency Syndromes. 2014;66(1):90-5.

9. Prior J, Burdge D, Maan E, Milner R, Hankins C, Klein M, et al. Fragility fractures and bone mineral density in HIV positive women: A case-control population-based study. Osteoporosis International. 2007;18(10):1345-53.

10. Sharma A, Shi Q, Hoover DR, Anastos K, Tien PC, Young MA, et al. Increased fracture incidence in middle-aged HIV-infected and HIV-uninfected women: Updated results from the women's interagency HIV study. Journal of Acquired Immune Deficiency Syndromes. 2015;70(1):54-61.

11. Triant VA, Brown TT, Lee H, Grinspoon SK. Fracture prevalence among human immunodeficiency virus (HIV)-infected versus non-HIV-infected patients in a large U.S. healthcare system. Journal of Clinical Endocrinology and Metabolism. 2008;93(9):3499-504.

12. Yin MT, McMahon DJ, Ferris DC, Zhang CA, Shu A, Staron R, et al. Low bone mass and high bone turnover in postmenopausal human immunodeficiency virus-infected women. Journal of Clinical Endocrinology and Metabolism. 2010;95(2):620-9.

Table 4. Quality assessment of included studies

|  |  | **Quality assessment criteria** | | | |
| --- | --- | --- | --- | --- | --- |
| **Author** | **Study design** | **Selection** | **Comparability** | **Outcome/ exposure** | **Overall quality** |
| Arnsten et al.^(3)^ 2007 | Cohort | **** | * | *** | 8 |
| Guaraldi et al.^(4)^  2011 | Case-control | **** | * | ** | 7 |
| Güerri-Fernandez et al.^(5)^ 2013 | Cohort | *** | ** | ** | 7 |
| Hansen et al.^(6)^ 2012 | Cohort | *** | * | **** | 8 |
| Peters et al.^(7)^ 2013 | Case-control | *** | * | ** | 6 |
| Prieto-Alhambra et al.^(8)^ 2014 | Case-control | *** | * | *** | 6 |
| Prior et al.^(9)^ 2007 | Case-control | *** | * | - | 4 |
| Sharma et al.^(10)^ 2015 | Cohort | ** | * | ** | 5 |
| Triant et al.^(11)^  2008 | Case-control | *** | * | - | 4 |
| Yin et al.^(12)^ 2010 | Cross-sectional | *** | * | *** | 7 |

Newcastle-Ottawa Scale was used to assess the selection, comparability and exposure of the case-control study, while the selection, comparability and outcome for the cohort study. -: no point; *: one point; **: two points; ***: three points.

Figure 2. PRISMA 2009 Flow Diagram of the studies included in the meta-analysis

Figure 3. Funnel plot for the studies included in the meta-analysis. The p-value for the Begg test was 0.420
